# Protective Effect of Fluorofenidone Against Acute Lung Injury Through Suppressing the MAPK/NF-κB Pathway

**DOI:** 10.3389/fphar.2021.772031

**Published:** 2021-12-20

**Authors:** Xin Lv, Tingting Yao, Rongling He, Yijun He, Mengyu Li, Yuanyuan Han, Yan Zhang, Lingzhi Long, Guoliang Jiang, Xiaoyun Cheng, Yanyun Xie, Ling Huang, Zhangzhe Peng, Gaoyun Hu, Qianbin Li, Lijian Tao, Jie Meng

**Affiliations:** ^1^ Department of Nephrology, Xiangya Hospital, Central South University, Changsha, China; ^2^ Department of Respirology, Third Xiangya Hospital, Central South University, Changsha, China; ^3^ Department of Respirology, Xiangya Hospital, Central South University, Changsha, China; ^4^ Organ Fibrosis Key Laboratory of Hunan Province, Changsha, China; ^5^ National International Collaborative Research Center for Medical Metabolomics, Changsha, China; ^6^ Faculty of Pharmaceutical Sciences, Central South University, Changsha, China

**Keywords:** fluorofenidone, acute lung injury, lipopolysaccharide, inflammation, apoptosis

## Abstract

Acute lung injury (ALI) is a severe disease that presents serious damage and excessive inflammation in lungs with high mortality without effective pharmacological therapy. Fluorofenidone (AKFPD) is a novel pyridone agent that has anti-fibrosis, anti-inflammation, and other pharmacological activities, while the effect of fluorofenidone on ALI is unclarified. Here, we elucidated the protective effects and underlying mechanism of fluorofenidone on lipopolysaccharide (LPS)-induced ALI. In this study, fluorofenidone alleviated lung tissue structure injury and reduced mortality, decreased the pulmonary inflammatory cell accumulation and level of inflammatory cytokines IL-1β, IL-6, and TNF-α in the bronchoalveolar lavage fluid, and attenuated pulmonary apoptosis in LPS-induced ALI mice. Moreover, fluorofenidone could block LPS-activated phosphorylation of ERK, JNK, and P38 and further inhibited the phosphorylation of IκB and P65. These results suggested that fluorofenidone can significantly contrast LPS-induced ALI through suppressing the activation of the MAPK/NF-κB signaling pathway, which indicates that fluorofenidone could be considered as a novel therapeutic candidate for ALI.

## Introduction

Acute lung injury (ALI) is a life-threatening pulmonary syndrome with high incidence and mortality worldwide and is also the most serious form of lung injury caused by the current pandemic coronavirus disease 2019 (COVID-19) (Peck and Hibbert, 2019; Peritore et al., 2021). The leading causes of ALI include bacterial infection, acid inhalation, fatty embolism, or virus infection. The main characteristics of ALI are excessive pulmonary inflammatory cell infiltration, diffuse alveolar damage, pulmonary epithelial cell apoptosis, and pulmonary edema, which led to reduction in tissue oxygenation and respiratory failure (Zhang et al., 2016; Silva et al., 2020). Until now, no satisfactory pharmacological therapies have been approved to lower ALI patients’ mortality. Herein, developing an effective, preventive, and therapeutic drug to improve clinical prognosis is necessary.

The pathophysiological mechanism of ALI is complex; it is likely that inflammation and apoptosis play a critical pathogenic role (Xu et al., 2015; Du et al., 2020). In ALI, inflammatory cells such as macrophages would accumulate and be activated in the lung. Then, pro-inflammatory cytokines, such as interleukin (IL)-1β, IL-6, and tumor necrosis factor-α (TNF-α), are produced and released by inflammatory cells and ultimately participate in the subsequent inflammatory cascade response (Akira et al., 1990; Wu et al., 2015; Robb et al., 2016). The consecutive inflammatory response would cause alveolar epithelial cell (AEC) damage and apoptosis and lead to pulmonary structure disorder and function loss (Ma et al., 2010; Wang et al., 2017). Nuclear factor-κB (NF-κB) and mitogen-activated protein kinases (MAPK) have been considered as target molecular pathways for ALI and acute respiratory distress syndrome (ARDS), which are also the essential active pathways for macrophages to produce inflammatory cytokines, and in turn to promote the development of ALI (Li Q. et al., 2020; Wang et al., 2020). Inhibiting the activation of the NF-κB/MAPK pathway would ameliorate pulmonary damage and reduce mortality of ALI mice induced by LPS (Zhang et al., 2018).

Fluorofenidone [1-(3-fluorophenyl)-5-methyl-2-([1H])-pyridone; AKFPD] is a novel pyridone anti-fibrosis compound which has demonstrated a remarkable therapeutic effect on organ fibrosis, including the kidney, liver, and lungs (Song et al., 2016; Dai et al., 2020; Tu et al., 2021). Currently, fluorofenidone is undergoing phase II clinical trial for liver fibrosis. Moreover, it shows a protective effect against acute kidney injury in several models, such as ischemia-reperfusion–induced renal injury (Jiang et al., 2019). In previous studies, fluorofenidone can reduce extracellular matrix deposition and inflammatory response in bleomycin-induced pulmonary fibrosis mice (Song et al., 2016). However, it remains unclear whether fluorofenidone has benefits in ALI therapy.

In this study, we first investigate the protective effect of AKFPD against ALI induced by LPS and the related underlying mechanisms.

## Methods

### Materials and Reagent

Fluorofenidone (AKFPD) was synthesized by Haikou Pharma (Haikou, China). LPS (*Escherichia coli O111:B4*) was purchased from Sigma Aldrich (#L2630). The antibody against cleaved caspase-3 (#9664), BCL-2 (#2876), Bax (#2772), phospho-ERK (#4370), phospho-JNK (#4668), phospho-P38 (#4631), ERK (#4695), JNK (#9252), P38 (#8690), phospho-IKK (#2697), phospho-IκB (#2859), IKK (#2682), IκB (#9242), P65 (#4764), anti-mouse IgG HRP-linked antibody (#7076), and anti-rabbit IgG HRP-linked antibody (#7074) were purchased from Cell Signaling Technology. The antibody against phospho-P65 (#ab76302) was from Abcam. The antibody against α-tubulin was purchased from proteintech (#66031-1-Ig). The F4/80 antibody was from BD bioscience (#565409), and the MPO antibody was from proteintech (#22225-1-AP). The BCA Protein Assay kit was from Thermo Fisher Scientific (#23225). A terminal deoxynucleotidyl transferase–mediated nick end labeling (TUNEL) kit was purchased from Roche (#11684817910). An FITC annexin V and propidium iodide (PI) staining kit was purchased from BD Biosciences (#556547). An ELISA test kit of IL-1β (#88-7013-88), TNF-α (#88-7324-22), and IL-6 (#88-7064-22) was purchased from Thermo Fisher Scientific. All other chemicals were of analytical grade.

### Acute Lung Injury Mouse Model

C57/BL mice were purchased from SJA Laboratory Animal Company (Hunan, China) and housed in a specific pathogen-free facility with a light/dark cycle of 12 h/12 h and randomly assigned to four groups: the control group, LPS group, AKFPD preventive group, and AKFPD treatment group (*n* = 6 per group). ALI models were generated according to previous studies (Ortiz-Munoz et al., 2014; Li T. et al., 2020). After anesthesia with 1% pentobarbital sodium, the mice were injected intratracheally with LPS (5 mg/kg) in 50 μl saline. The control group was treated with saline at the same volume. The AKFPD preventive group was administered orally with AKFPD 2 days (500 mg/kg/day) before LPS injection. The AKFPD treatment group was administered orally with AKFPD (500 mg/kg/day) 1 h after LPS injection for 2 days. LPS group were administered with the same volume of vehicle. Following a duration of 48 h after LPS injection, mice were sacrificed for subsequent assays.

To observe the effect of fluorofenidone on survival, 36 mice were randomly divided into three groups: the control group, LPS group, and AKFPD preventive group (*n* = 12 per group). Survival was assessed every 6 h. All experimental protocols were performed according to the guidelines of the National Institutes of Health and Central South University, and all procedures were approved by the Institutional Animal Care and Use Committee of Central South University.

### Histopathology and Immunohistochemistry

Lung tissue samples were fixed in 4% paraformaldehyde for 2 days and then were embedded in paraffin. For the histological examination, the samples were cut into 4 μm sections and stained with hematoxylin and eosin (H&E). The stained slides were analyzed with a light microscope under identical conditions. The sections were examined at 200× magnification for all the groups. Histopathology was scored using a semi-quantitative scale from 0 (normal and no focal inflammatory infiltrates) to 4 (severe infiltration and damage in the lung structure), as described before (Barriga et al., 2021).

Immunohistochemistry for F4/80 and myeloperoxidase (MPO) was performed, as previously described (Liao et al., 2021). After deparaffinization and rehydration, the lung tissue slides were treated with 3% H_2_O_2_ for 20 min, denatured for 15 min in boiling 10 mM citric acid (pH 6.0), blocked with 5% bovine serum albumin for 30 min at room temperature, and then incubated overnight at 4°C with the F4/80 antibody or MPO antibody. After washing with PBS and incubation with secondary antibodies, the samples were visualized by diaminobenzidine staining. A total of 10 scopes were randomly selected in each sample under 200-fold magnification for quantitative analysis.

### Bronchoalveolar Lavage Fluid Collection

BALF was collected by lavaging the lungs with 0.5 ml precooled phosphate buffered saline three times in all groups. The supernatant of BALF was obtained after centrifugation at 3,000 g for 15 min at 4°C and stored at −20°C for further analysis. The total protein concentration in the supernatant was measured using the BCA Protein Assay kit.

### Cell Culture and Treatment

Mouse lung epithelial cells (MLE-12) and immortalized mouse bone marrow–derived macrophages (iBMDMs) were used. MLE-12 cells were incubated in Dulbecco’s modified Eagle’s medium (DMEM)/F12; iBMDMs were incubated in Roswell Park Memorial Institute 1640 (RPMI 1640) medium. Media were supplemented with 10% fetal bovine serum, 100 U/ml penicillin, and 100 U/ml streptomycin. Cells were maintained at 37°C under humidified 5% CO_2_.

For NF-κB/MAPK pathway evaluation, iBMDMs were stimulated with LPS (1 μg/ml) for 0.5–3 h, and AKFPD (400 μg/ml) were added 24 h before LPS treatment. For apoptosis level detection, MLE-12 were stimulated with LPS (1 μg/ml) for 24 h, and AKFPD (400 μg/ml) were added 24 h before LPS treatment. Each experiment was replicated at least three times.

### ELISA

Concentrations of IL-6, IL-1β, and TNF-α of BALF and the cell supernatant were detected by using the ELISA kit according to manufacturer’s instructions.

### TUNEL Assay

TUNEL assay was performed in 4 μm paraffined sections with the *In Situ* Cell Death Detection Kit, fluorescein, according to the manufacturer’s instructions. After deparaffinization and rehydration, lung slides were denatured in 10 mM citric acid buffer, following by blocking in 3% goat serum. Reagent 1 (TDT) and reagent 2 (dUTP) were mixed at 1:10 and added to the slices at 4°C overnight. After washing with PBS, slides were stained with DAPI for 10 min. A total of five scopes were randomly selected in each sample under 200-fold magnification for quantitative analysis.

### Immunofluorescence

After deparaffinization and rehydration, lung slides were denatured for 15 min in boiling 10 mM citric acid (pH 6.0) and blocked with 5% bovine serum albumin for 30 min at room temperature and then incubated overnight at 4°C with the P65 antibody. After washing with PBS, slides were incubated with secondary antibodies for 1 h and then were stained with DAPI for 10 min.

### Flow Cytometric Analysis

MLE-12 cells were seeded on 6-well culture plates in a complete medium containing 10% FBS for 24 h. Wells were randomly separated into three groups: control, LPS, and LPS + AKFPD. The LPS + AKFPD group was pretreated with AKFPD (400 μg/ml) for 24 h. After AKFPD pretreatment, the LPS group and LPS + AKFPD group were incubated with LPS (1 μg/ml) for 24 h to induce cell apoptosis. After that, cells were harvested, stained with the FITC Annexin V and PI staining kit according to the manufacturer’s instructions, and analyzed by flow cytometry to identify cell apoptosis. Annexin V^+^ and PI^−^ cells were considered as early apoptotic cells, and annexin V^+^ and PI^+^ cells were considered as late apoptotic cells. The experiment was replicated at least three times.

### Western Blot Analysis

Protein concentrations were determined using the BCA Protein Assay Kit. For Western blot analysis, 20–40 μg of protein was separated on sodium dodecyl sulfate–polyacrylamide (SDS-polyacrylamide) gel followed by transfer to polyvinylidene difluoride membranes. The membranes then were blocked in 5% skim milk for 1 h at room temperature and were incubated overnight at 4°C with the primary antibodies against p-IKK/IKK (1:1000), p-IκB/IκB (1:1000), p-P65/P65 (1:1000), p-ERK/ERK (1:1000), p-P38/P38 (1:1000), p-JNK/JNK (1:1000), caspase-3 (1:1000), Bax (1:1000), Bcl-2 (1:1000), and α-tubulin (1:5000). Then, the membranes were subsequently incubated with HRP-conjugated secondary antibodies for 1 h at room temperature. The bands were visualized by the ECL substrate and analyzed using ImageJ software.

### Statistical Analysis

Results were presented as the mean value ±standard deviation. One-way analysis of variance (ANOVA) followed by Tukey’s multiple comparison tests with Prism software (version 6.01; GraphPad, United States) was used for comparison among all different groups. The log-rank test was used to compare the survival rate. *p* value <0.05 was considered as statistically significant.

## Results

### Fluorofenidone Treatment Ameliorated Lung Injury and Mortality in the LPS-Induced ALI Mouse Model

**FIGURE 1:**
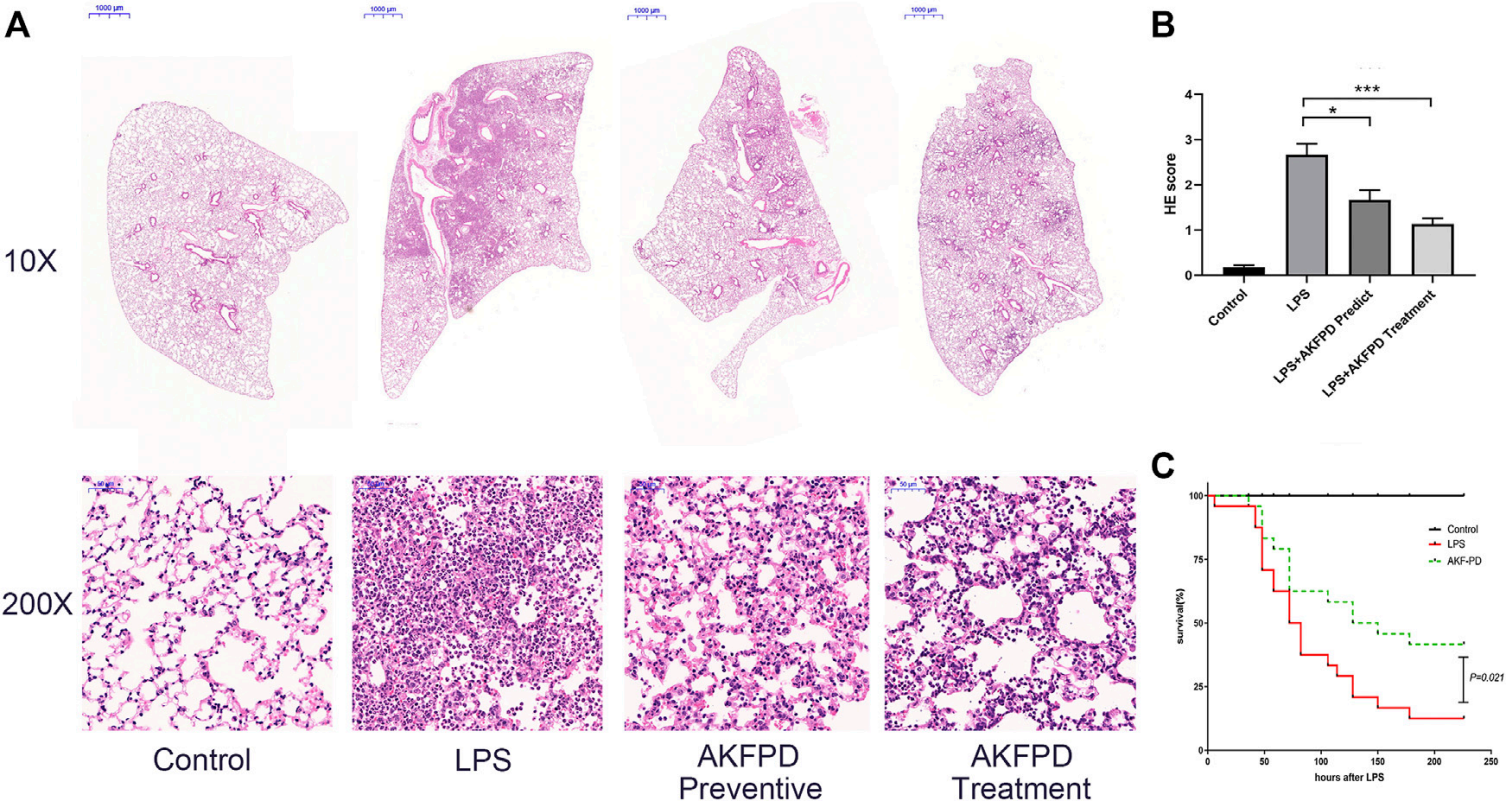
Fluorofenidone treatment ameliorated lung injury and mortality in the LPS-induced ALI mouse model. An acute lung injury model was established by LPS (5 mg/kg, intratracheal injection). The lung tissue was collected 48 h after the LPS challenge. AKFPD was administered orally to observe the preventive and therapeutic effect (*n* = 6 per group). **(A)** Representative images of HE staining of the pathological changes in lung tissues from different groups (10× magnification, scale bar = 1000 μm and 200 × magnification, scale bar = 50 μm). **(B)** The lung injury scores of HE staining for pulmonary damage. Each unit randomly chose 10 scopes under 200 × magnification to calculate the average values. **(C)** Analysis of the survival rate of ALI mice induced by LPS (*n* = 12 per group). Data were represented as mean ± SD. *p < 0.05 and ***p < 0.005 vs. LPS group.

To determine whether fluorofenidone has a protective effect on ALI, we evaluated the histopathologic feature of the lungs in the LPS-induced ALI mouse model. As showed in H&E staining (Figure 1A), LPS administration induced extensive and serious inflammation with massive inflammatory cell infiltration, alveolar architecture disorder, and partial consolidation, while the control group showed a normal pulmonary architecture. Compared with the LPS group, fluorofenidone treatment remarkably relieved those pathological symptoms and decreased lung injury scores (Figure 1B). In order to confirm the late protective effect of fluorofenidone in ALI, we further observed the survival rate in ALI mice, and the results showed that treatment with fluorofenidone remarkably reduced LPS-induced mortality in ALI mice (Figure 1C).

### Fluorofenidone Decreased the Pulmonary Inflammation in the LPS-Induced Mouse Model

**FIGURE 2:**
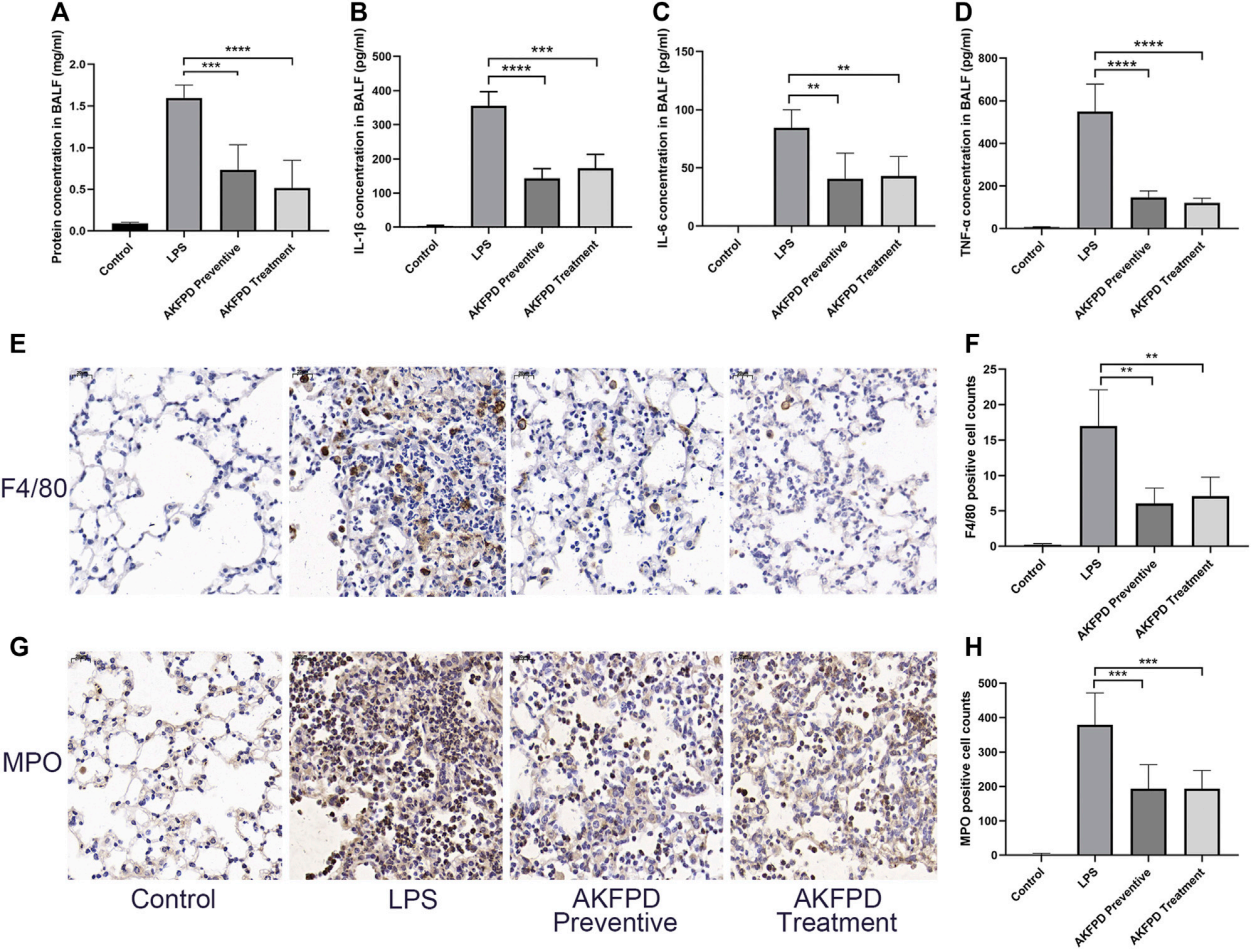
Fluorofenidone decreased the pulmonary inflammation in the LPS-induced mouse model. **(A)** Concentration of total protein in BALF. **(B–D)** Expression of IL-1β, IL-6, and TNF-α levels in the BALF of mice. **(E–H)** Immunohistochemical staining of F4/80, MPO, and positive cell count analysis in the lung tissue of different groups of mice. Scale bar = 20 μm. Data were represented as mean ± SD. *n* = 6 per group. **p < 0.01, ***p < 0.005, and ****p < 0.001 vs. LPS group.

It is well known that inflammation plays a critical role in the development of ALI. Numerous pro-inflammatory cytokines and chemokines would be released to damage pulmonary epithelial cells and bronchial epithelial cells and further recruit inflammatory cells such as macrophages, to induce the inflammatory cascade response (Ma et al., 2010; Wang et al., 2017). To further evaluate the effects of fluorofenidone on ALI, we investigated the inflammatory level in BALF of ALI mice. We found that fluorofenidone treatment significantly diminished the elevated levels of IL-1β, TNF-α, and MCP-1 in BALF of LPS-induced ALI mice (Figures 2A–C). Moreover, the percentage of inflammatory cells in the lungs was determined by immunohistochemistry. As showed in Figures 2E–H, fluorofenidone administration obviously decreased the macrophage marker F4/80 and neutrophil marker MPO positive cell counts in the lungs of mice. These results suggest that fluorofenidone can interrupt the LPS-induced inflammatory cascade and reduce the inflammatory response in ALI.

### Fluorofenidone Decreased Mitochondrial Apoptosis in the Lungs of the LPS-Induced Mouse Model

**FIGURE 3:**
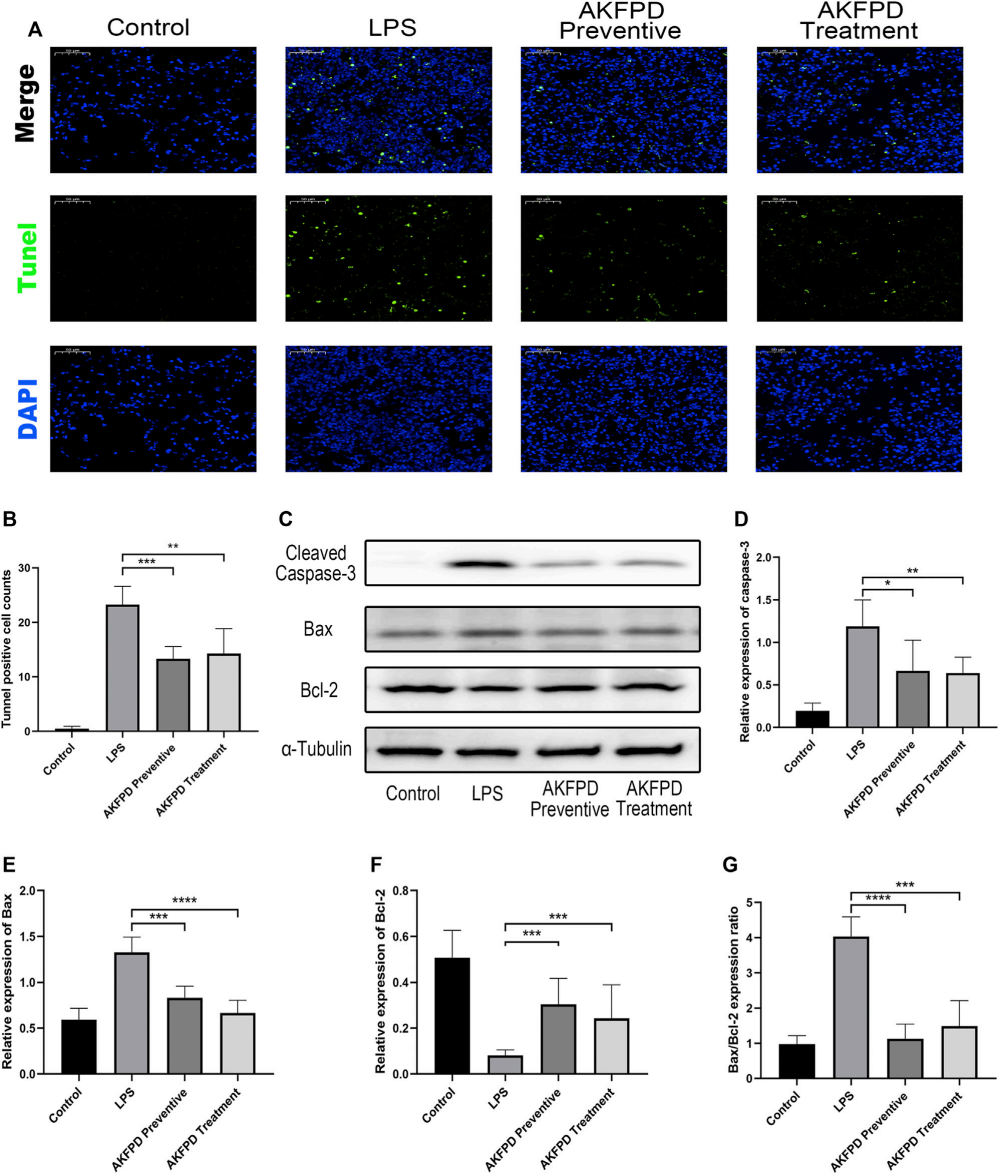
Fluorofenidone decreased the pulmonary inflammation in the LPS-induced mouse model. **(A,B)** Representative images of TUNEL staining of lung tissues and positive cell count analysis of different groups. Green represents TUNEL positive cells, and blue represents the nucleus. Scale bar = 50 μm. **(C)** Western blot and analysis of cleaved caspase-3, Bax, and Bcl-2 in the lung tissue. **(D–G)** Quantification of cleaved caspase-3, Bax, Bcl-2, and the ratio of Bax to Bcl-2 in the lung tissue. Data were represented as mean ± SD. *n* = 6 per group. *p < 0.05, **p < 0.01, ***p < 0.005, and ****p < 0.001 vs. the LPS group.

Previous studies have revealed that excessive AEC apoptosis is observed in LPS-induced ALI (Yanagihara et al., 2001; Luo et al., 2019). AEC apoptosis could be mediated by various pathways, such as the mitochondrial dysregulation–mediated pathway or DNA damage-mediated pathway (Galani et al., 2010). Inhibition of pulmonary epithelial apoptosis would improve the pulmonary function and survival rate of ALI mice (Gong et al., 2017). Thus, our study further investigated whether fluorofenidone could reduce cell apoptosis in ALI mice. The results showed that fluorofenidone markedly reduced the number of TUNEL positive cells in the lungs of ALI mice induced by LPS (Figures 3A,B). Furthermore, we found that fluorofenidone could significantly increase the expression levels of the mitochondrial anti-apoptosis factor B-cell lymphoma 2 (Bcl-2), decrease the pro-apoptosis factor Bcl-2 X-associated protein (Bax), the ratio of Bax to Bcl-2, and cleaved caspase-3 in the lung tissue of ALI mice (Figures 3C–G), which suggests that fluorofenidone could ameliorate the mitochondrial-mediated apoptosis in LPS-induced ALI mice.

### Fluorofenidone Suppressed NF-κB/MAPK Pathway Activation in LPS-Induced ALI Mice

**FIGURE 4:**
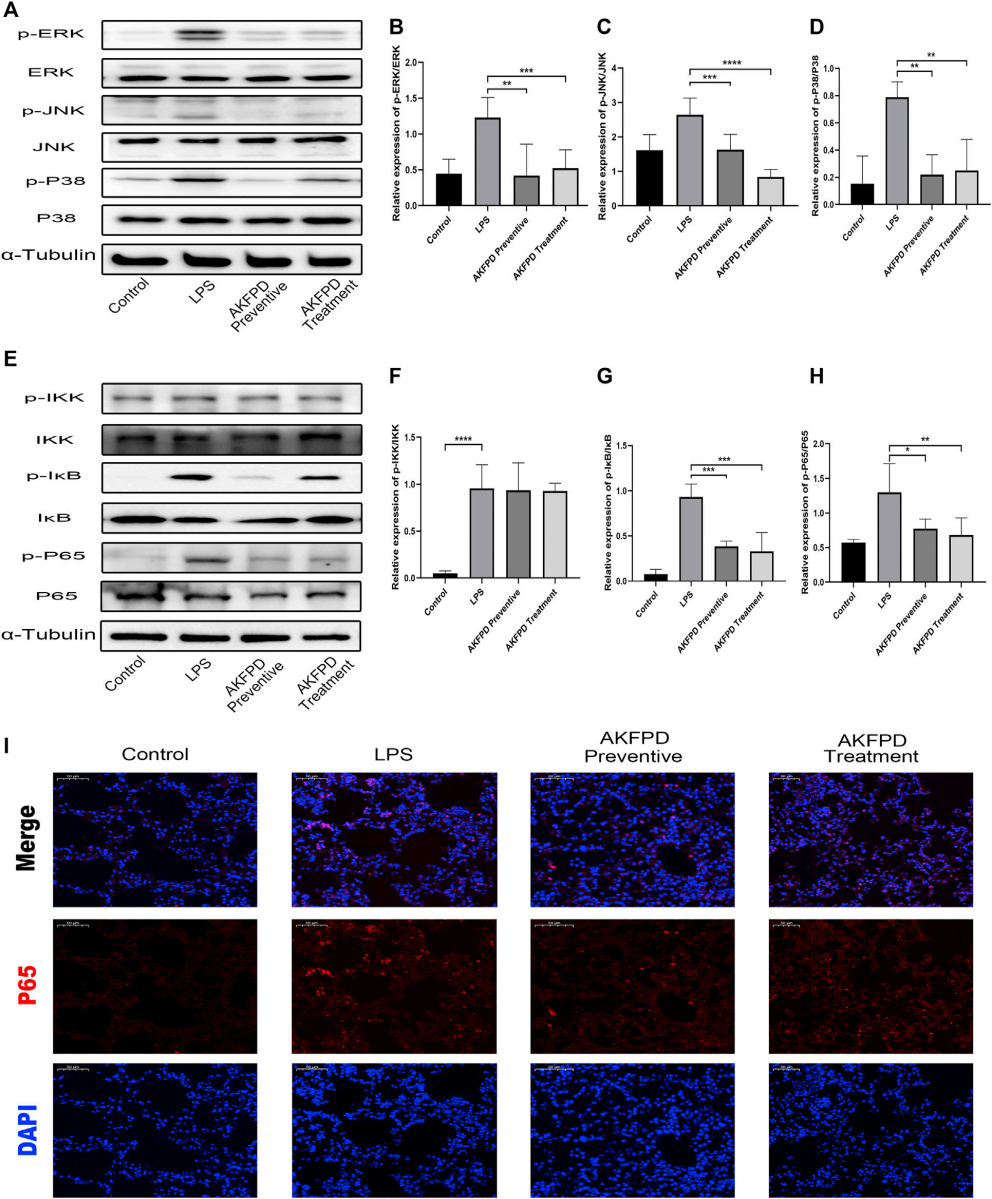
Fluorofenidone suppressed NF-κB/MAPK pathway activation in LPS-induced ALI mice. Western blot **(A)** and quantitative analysis of p-ERK, **(B)** p-JNK, **(C)** and p-P38 **(D)** in the lung tissue of different groups of mice. Western blot **(E)** and quantitative analysis of p-IKK, **(F)** p-IκB, **(G)** and p-P65 **(H)** in the lung tissue of mice. **(I)** Representative images of immunofluorescence staining of P65 in the lung tissue. Scale bar = 50 μm. Data were represented as mean ± SD. *n* = 6 per group. *p < 0.05, **p < 0.01, ***p < 0.005, and ****p < 0.001 vs. the LPS group.

It has been reported that LPS can induce the production and secretion of various inflammatory mediators by stimulating the NF-κB pathway and MAPK pathways (Guo et al., 2018). Hence, we further explored whether fluorofenidone exerts its inhibitory effect on NF-κB and MAPK signaling pathway in LPS-induced ALI mice. In our study, the expressions of phosphorylated ERK, JNK, and P38 in the LPS group were remarkably increased compared with those of the control group, while fluorofenidone administration significantly reduced those changes (Figures 4A–D). Moreover, we also found that fluorofenidone remarkably suppressed phosphorylation of IκB and NF-κB induced by LPS in lung tissues, but the expression of phosphorylated IKK was not affected (Figures 4E–H). Meanwhile, immunofluorescence also showed that fluorofenidone obviously prevented nuclear translocation of NF-κB in the lungs of ALI mice (Figure 4I).

### Fluorofenidone Ameliorated Mitochondrial-Mediated Apoptosis *In Vitro*

**FIGURE 5:**
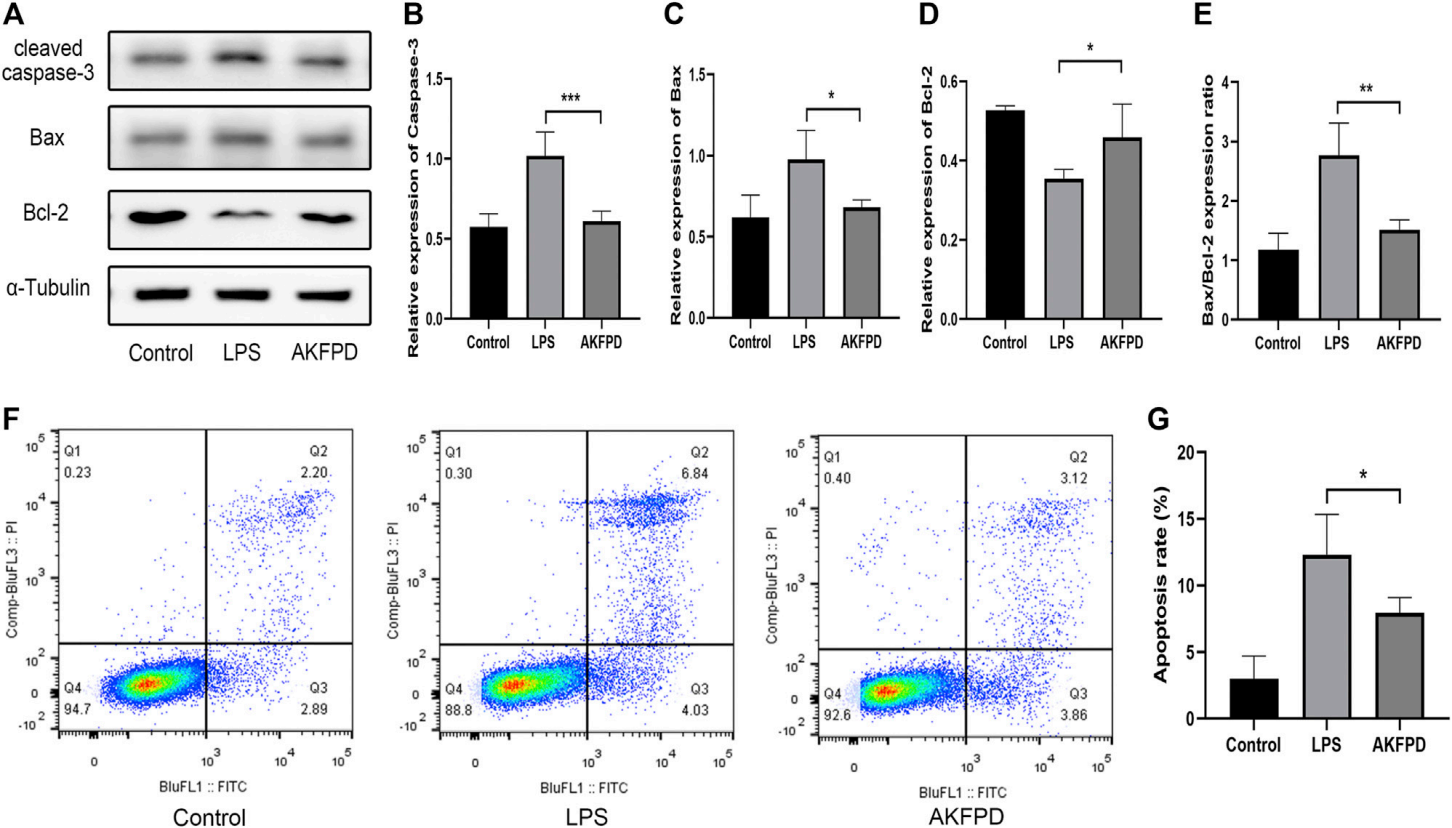
Fluorofenidone ameliorated mitochondrial-mediated apoptosis *in vitro*. Western-blot **(A)** and quantitative analysis of cleaved caspase-3, **(B)** Bax, **(C)** Bcl-2, **(D)** and the ratio of Bax to Bcl-2 **(E)** in different groups of MLE-12 cells. **(F,G)** Flow cytometry of FITC/PI and quantitative comparison of MLE-12 cells stimulated by LPS (1 μg/ml) with/without AKFPD (400 μg/ml) pretreatment. Data were represented as mean ± SD. *n* = 4 per group. *p < 0.05 and ***p < 0.005 vs. the LPS group.

To further validate the effects of fluorofenidone on apoptosis, we mimicked LPS-induced lung injury in mouse intrinsic alveolar epithelial cell lines (MLE-12) by treating cells with LPS. The results of Western blot showed that LPS treatment stimulated the expression of Bax and cleaved caspase-3, while decreased that of Bcl-2 and increased the ratio of Bax to Bcl-2. However, pretreatment with fluorofenidone markedly diminished those changes (Figures 5A–E). Meanwhile, we measured the proportion of apoptotic cells by flow cytometry assay. As shown in Figures 5F,G, more apoptotic cells were observed in LPS-induced MLE-12 cells, while fluorofenidone pretreatment significantly reduced the proportion of apoptotic alveolar epithelial cells.

### Fluorofenidone Inhibited Inflammation by Suppressing the Activation of the NF-κB/MAPK Pathway *In Vitro*

**FIGURE 6:**
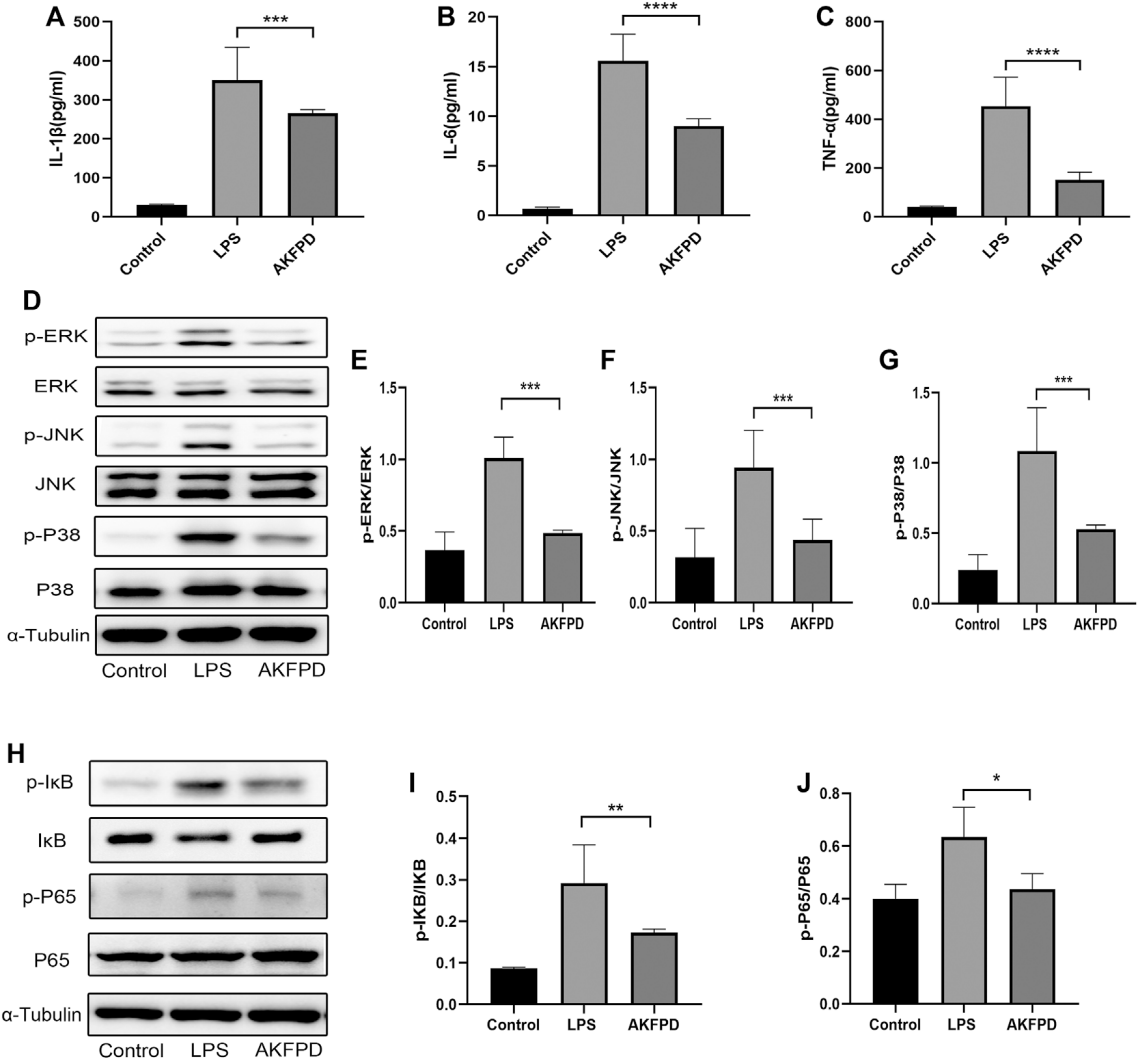
Fluorofenidone inhibited inflammation by suppressing activation of the NF-κB/MAPK pathway *in vitro*. **(A–C)** ELISA analysis of IL-1β, TNF-α, and IL-6 in the supernatant of BMDMs in different groups. Western blot **(D)** and quantitative analysis of p-ERK **(E)**, p-JNK **(F)**, and p-P38 **(G)** proteins in BMDMs. Western blot **(H)** and quantitative analysis of pIκB **(I)** and p-P65 **(J)** in BMDMs. Data were represented as mean ± SD. *n* = 4 per group. *p < 0.05, **p < 0.01, ***p < 0.005, and ****p < 0.001 vs. the LPS group.

It is widely accepted that the inflammatory cascade response in ALI majorly occurs and initiates in macrophages, including the inflammatory signaling pathway activation and the proinflammatory factor release (Zhang et al., 2016). Therefore, we determined the anti-inflammatory effects of fluorofenidone in BMDMs. As shown in Figure 6, preincubation with fluorofenidone markedly reduced the expression levels of IL-1β, TNF-α, and IL-6 in the supernatant of BMDMs elevated by LPS (Figures 6A–C). Moreover, the effect of fluorofenidone on NF-κB/MAPK pathway activation on LPS-induced BMDMs was detected. The results of Western blot showed that compared with the control group, the phosphorylation of IκB, NF-κB, ERK, JNK, and P38 was significantly increased upon LPS stimulation, and pretreatment with fluorofenidone markedly reversed those effects (Figures 6D–J). Those results suggest that fluorofenidone could alleviate the LPS-induced inflammation response *via* suppressing NF-κB/MAPK pathway activation in macrophages.

## Discussion

Acute lung injury is a serious pulmonary disease induced by various factors, which could develop to fatal complication and ARDS. Unfortunately, there is still lack of effective therapeutic medicine. As the me-better drug of pirfenidone, fluorofenidone has a higher no observed adverse effect level (NOAEL) and longer half-time period than pirfenidone and has shown obviously anti-fibrosis effects (Song et al., 2016; Dai et al., 2020; Tu et al., 2021). In the present study, we not only proved that fluorofenidone could efficiently protect ALI for the first time but also demonstrated that fluorofenidone could provide preventive and therapeutic effects for ALI.

Inflammatory cell infiltration and pro-inflammatory cytokine production are both significant features in the development of LPS-induced ALI; uncontrolled inflammatory responses are regarded as the key promoter of ALI pathogenesis (Martin 1997; Goodman et al., 2003). The network of inflammatory cytokines, such as TNF-α, IL-6, and IL-1β, has been shown to be involved in the initiation of ALI (Bhatia and Moochhala, 2004). The levels of those cytokines in plasma or BALF have been proved as early biomarkers of lung injury and predictive factors of mortality in ARDS (Remick et al., 2005). Those activated cytokines form a network, participating in the recruitment of inflammatory cells to the injury site and initiating a chain reaction which further induces the escalation of inflammation, damage the normal pulmonary structure, and aggravate the severity of the disease (Medzhitov 2008). Although the injury induced by LPS could not completely duplicate the pulmonary pathological change stimulated by SARS-CoV2, LPS-induced cytokines storm and the inflammatory cascade could still be helpful to screen therapeutic candidates for COVID-19. In fact, the level of IL-6 was confirmed to be correlated with pulmonary infiltration areas of severe COVID-19 patients (Chen et al., 2020). In this study, our data indicated that fluorofenidone was able to inhibit the LPS-induced pulmonary diffuse inflammatory response by reducing levels of inflammatory cytokines and alleviating the infiltration of inflammatory cells such as macrophages and neutrophils. In addition, fluorofenidone significantly attenuated pulmonary pathological changes and reduced mortality of ALI mice, which suggested that fluorofenidone was protective against ALI through its significant anti-inflammation effect. As a novel compound that is under clinical trial, fluorofenidone is promising for clinical application for ALI induced by various factors. At present, glucocorticoid is still the main therapeutic choice for the inflammatory storm of COVID-19 in clinics, but the related side effects (such as immunosuppression) are serious, which would be avoided by fluorofenidone, suggesting that it may be a candidate drug for the treatment of lung injury of COVID-19.

Apoptosis is a process of programmed cell death, occurs in multicellular organisms, and participates in mandating cell homeostasis (Jia et al., 2018). Excessive AEC apoptosis is one of the pivotal characteristics of ALI (Yang et al., 2020). LPS can induce cell apoptosis by inducing inflammatory response and mitochondrial damage. During this process, the pro-apoptosis factor Bax would induce pores formed in the damaged mitochondrial membrane, resulting in the change of mitochondrial membrane permeability, which however could be blocked by the anti-apoptosis factor Bcl-2 (Li et al., 2018). Apoptogenic factors would be released from the mitochondria and drive apoptosome to activate caspase-9, which further cleaves caspase-3 to initiate a cell death program. Hence, the ratio of Bax to Bcl-2 is considered as a pivotal factor to determine cell apoptosis or survival (Upadhyay et al., 2003; Estaphan et al., 2020). Notably, inhibition of intrinsic apoptosis can markedly ameliorate the lung damage induced by SARS-CoV-2, suggesting that this process can be targeted to attenuate severity of COVID-19 (Chu et al., 2021). In the present study, we proved that fluorofenidone significantly reduced the expression of cleaved caspase-3, Bax, and restored the expression of Bcl-2, diminished the elevated ratio of Bax to Bcl-2, and further decreased the numbers of apoptotic cells *in vivo* and *in vitro*. These data indicated that the protective effect of fluorofenidone may be partially owing to its anti-apoptosis effect.

Extensive evidence has shown that activation of the MAPK and NF-κB pathway promotes inflammation and programmed cell death in ALI induced by LPS (He et al., 2019; Pooladanda et al., 2019). The MAPK pathway activated in response to extracellular stimuli and regulated the synthesis and release of pro-inflammatory mediators. Inhibition of the phosphorylation of the MAPK pathway can obviously attenuate the pro-inflammatory mediator transcript and release (Zhang et al., 2021). The phosphorylated MAPK cascade would further lead to IκB-α activation, which induces the NF-κB activity (Ko et al., 2020). Meanwhile, IκB-α could be phosphorylated directly by LPS stimulation, following nuclear translocation of P65 then inducing inflammatory response (Guo et al., 2018). NF-κB is the critical factor relating to inflammation, apoptosis, and proliferation, which is considered as an ideal target to mediate the pro-inflammatory molecule expression in ALI (Huang et al., 2017; Jiang et al., 2017). In our study, we found that fluorofenidone could inhibit the phosphorylation of the MAPK pathway, including P38, JNK, and ERK, and further suppressed the activation of the NF-κB pathway both *in vivo* and *in vitro*, which suggested that the beneficial effect of fluorofenidone in ALI may be attributed to the blocking of MAPK and NF-κB pathways. Regrettably, we did not use inhibitors of MAPK or NF-κB pathways to further explore the therapeutic mechanism of fluorofenidone in ALI, which would be testified in our future work.

Taken together, our results indicate that fluorofenidone exerts its anti-inflammatroy and anti-apoptosis effects in ALI. The possible mechanisms are associated with the inactivation of MAPK and NF-κB pathways. As a novel compound that is under clinical trial, fluorofenidone is promising for clinical application and should be considered as a potential candidate drug for acute lung injury.

## Data Availability

The raw data supporting the conclusions of this article will be made available by the authors, without undue reservation.
